# Tattoo ink exposure is associated with lymphoma and skin cancers – a Danish study of twins

**DOI:** 10.1186/s12889-025-21413-3

**Published:** 2025-01-15

**Authors:** Signe Bedsted Clemmensen, Jonas Mengel-From, Jaakko Kaprio, Henrik Frederiksen, Jacob von Bornemann Hjelmborg

**Affiliations:** 1 Department of Epidemiology, Biostatistics, and Biodemography, Institute of Public Health, University of Southern Denmark, Odense, Denmark; 2 Danish Twin Registry, Institute of Public Health, University of Southern Denmark, Odense, Denmark; 3 Department of Clinical Genetics, Odense University Hospital, Odense, Denmark; 4 Institute for Molecular Medicine Finland FIMM, HiLIFE, University of Helsinki, Helsinki, Finland; 5 Department of Haematology, Odense University Hospital, Odense, Denmark; 6 Department of Clinical Research, University of Southern Denmark, Odense, Denmark

**Keywords:** Tattooing, Lymphoma, Skin cancer, Cancer prevention, Public health

## Abstract

**Background:**

We aim to study the potential association between tattoo ink exposure and development of certain types of cancers in the recently established Danish Twin Tattoo Cohort. Tattoo ink is known to transfer from skin to blood and accumulate in regional lymph nodes. We are concerned that tattoo ink induces inflammation at the deposit site, leading to chronic inflammation and increasing risk of abnormal cell proliferation, especially skin cancer and lymphoma.

**Methods:**

We conducted two designs of twin studies to improve confounder control: A cohort study of 2,367 randomly selected twins and a case-control study of 316 twins born in the period 1960–1996. Cancer diagnoses (ICD-10) were retrieved from the Danish Cancer Registry and tattoo ink exposure from the Danish Twin Tattoo survey from 2021. The analysis addressed effects of time-varying exposure.

**Results:**

In the case-control study, individual-level analysis resulted in a hazard of skin cancer (of any type except basal cell carcinoma) that was 1.62 times higher among tattooed individuals (95% CI: 1.08–2.41). Twin-matched analysis of 14 twin pairs discordant for tattoo ink exposure and skin cancer showed HR = 1.33 (95% CI: 0.46–3.84). For skin cancer and lymphoma, increased hazards were found for tattoos larger than the palm of a hand: HR = 2.37 (95% CI: 1.11–5.06) and HR = 2.73 (95% CI: 1.33–5.60), respectively. In the cohort study design, individual-level analysis resulted in a hazard ratio of 3.91 (95% CI: 1.42–10.8) for skin cancer and 2.83 (95% CI: 1.30–6.16) for basal cell carcinoma.

**Conclusion:**

In conclusion, our study suggests an increased hazard of lymphoma and skin cancers among tattooed individuals, demonstrated through two designs: a twin cohort and a case-cotwin study. We are concerned that tattoo ink interacting with surrounding cells may have severe consequences. Studies that pinpoint the etiological pathway of tattoo ink induced carcinogenesis are recommended to benefit public health.

**Supplementary Information:**

The online version contains supplementary material available at 10.1186/s12889-025-21413-3.

## Background

In recent decades, the number of people getting tattooed have increased. The overall proportion of persons with tattoos (i.e. prevalence) is up to 20–25% in some countries and nearly twice as high among the younger generations [1–4]. With the increased popularity of tattoos, safety regarding tattooing and exposure to tattoo ink becomes increasingly relevant, and the lack of studies based on population epidemiologic data to assess carcinogenicity is of particular concern.

Particles from tattoo ink have been found to accumulate in regional lymph nodes [5–7], and they may be transported through the bloodstream to other organs [8–12]. It is an open question whether they could cause harm in the skin, to the immune system, and even to other internal organs.

The most frequently used tattoo ink is black. Black ink typically contains soot products like carbon black, which is listed as possibly carcinogenic to humans (mainly based on studies of carbon black inhalation and risk of lung cancer) by the International Agency for Research on Cancer (IARC) [13]. Through the incomplete combustion used for carbon black production, polycyclic aromatic hydrocarbons (PAHs) are formed as byproducts. One of the most dangerous of these is benzo[a]pyrene (BaP), which is classified as carcinogenic to humans by the IARC [14]. Another hazardous substance (typically appearing in colored inks) is azo compounds, as these may release carcinogenic aromatic amines following exposure to sunlight or laser treatment tattoo removal [15]. On a more general level, red ink has been observed as the most predominantly used color among patients with allergic reactions [16].

The long latency period of cancer and the possible combination of a variety of environmental exposures make it difficult to describe the origin of disease development. The lack of knowledge in this field has led several researchers and health-related organizations such as IARC and the European Commission to call for epidemiologic studies to investigate the proposed associations between exposure to tattoo ink and risk of certain cancers such as lymphoma and skin cancers [9, 17, 18]. To the best of our knowledge, there are only three publications of studies seeking to obtain empirical evidence regarding this potential link between tattoo ink and increased risk of cancer, specifically lymphoma [19, 20], multiple myeloma [19], and basal cell carcinoma [21]. The most recent study of lymphoma [20] provided evidence suggesting an increased risk among tattooed individuals, while the results from the study of basal cell carcinoma [21] were not statistically significant, but pointed toward increased odds among tattooed individuals. Thus, there is a deficiency in the existing literature, and, given the growing popularity of tattooing, there is an urgent need for more studies.

We conjecture that tattoo ink induces inflammation at the deposit site that may eventually lead to chronic inflammation and increased risk of abnormal cell proliferation, especially skin cancer and lymphoma. We emphasize that this may happen for any type of ink due to foreign body immunologic response. In addition, ink particles with known or suspected carcinogenic properties may gradually increase this risk over time. In this study, we aim to assess the possible relationship between tattoo ink exposure and development of certain types of cancer. The cancers considered are those related directly to the skin serving as the original deposit site of tattoo ink along with lymphoma according to the ink deposit conjecture and cancer of the bladder and urinary tract as a deposit site for ink particles transported through the bloodstream.

Our recently established Danish Twin Tattoo Cohort [22] provided the opportunity to study described hypothesis with elaborate confounder control through parallel analyses using individual-level analysis along with the matched case-cotwin design. For pairs in which one twin had been diagnosed with cancer and the other had not, we wanted to investigate which of the twins had been exposed to tattoo ink, as this approach might provide tentative insights into the processes governing ink exposures and cancer development, which are expected to occur over a long duration.

## Methods

The Danish Twin Tattoo Cohort (DTTC) was established in 2021, and it aimed to collect data for a case-cotwin study and a twin cohort [22]. Subsets of these samples are analyzed in this paper. The DTTC was based on a questionnaire survey about tattoo ink exposure among Danish twins and entitled *Risk factors of certain types of cancer diseases*. An overview of the questionnaire is provided in Supplementary Fig. 1. The full Danish questionnaire is available in the thesis by Clemmensen [22]. The survey was carried out from January to July 2021. Twins were identified through linkage of the nationwide Danish Twin Register (DTR) and the National Cancer Register using unique personal identification numbers. Cancers were categorized into main cancer types according to NORDCAN [23, 24] and thereby complying with Nordic cancer registration. The outcome cancers were a priori chosen to represent our ink deposit conjecture stated in the introduction. Hence, the cancer sites include areas where ink is known to deposit, such as the skin, lymph nodes [5–7], and internal organs, which the ink is suspected to reach through the bloodstream [8–12]. We note that both the case-cotwin design and the cohort study design deal with adult cancer survivors as explained in the following paragraphs. A full description of the cohort compilation was made by Clemmensen [22].

The survey included questions about tattoo status, age at first tattoo, colors, and size (measured in estimated units of the size of the palm of one’s hand). Additionally, there were questions about potential confounders and known cancer risk factors such as smoking, physical exercise, alcohol consumption, and education.

### The case-cotwin study

The twin pairs included in the case-cotwin study were identified as all twin pairs born in Denmark from 1960 to 1996, i.e., all twins had reached age 20 years at end of follow-up (January 1, 2017). Inclusion criteria were: (1) At least one twin had been diagnosed with one (or more) of the following cancers after reaching age 20 years: Hodgkin or non-Hodgkin lymphoma (ICD-10 was used along with ICD-O-3 by NORDCAN when identifying lymphoma incident cases), skin cancer (melanoma ICD-10: C43 and non-melanoma ICD-10: C44 – excluding basal cell carcinoma ICD-10: C44.91) and bladder/urinary tract cancer. (2) At least one twin could be contacted, i.e. was alive, not emigrated, and had not waived contact by researchers. A total of 568 individual twins complied with the first criteria, and 504 individual twins (including both twins in 219 pairs) further complied with the second criteria and were invited to participate in the survey. Of these, 316 (56%) responded.

### The twin cohort

The cohort study included 2,459 twin pairs born 1960–1996, randomly selected among all pairs from the DTR, where at least one twin could be contacted. A total of 4,532 individual twins were invited and 2,367 (52%) participated (including both twins in 673 pairs). In the twin cohort, we considered lymphoma, skin cancer, and basal cell carcinoma as outcomes. The lymphoma and skin cancer cases and their cotwins were a subset of the case-cotwin study.

### Statistical analysis

Frequency and percentage or median and interquartile range (IQR) are provided to describe the case-cotwin study along with tattoo size, whether certain colors of ink were used, and smoking habits. Similar information (along with other information) was previously reported for the twin cohort [4]. Only measured confounders that were known at the time of exposure were included. Fewer than five twins had multiple diagnoses; for cases of the same type (e.g. skin cancer), only time to first diagnosis was used, thus disregarding subsequent diagnoses. For cases involving different types (e.g. lymphoma and skin cancer), the individual was included as a case in both respective samples.

### Time-to-event analysis

As time to event of cancer is expected to confound the studied relationship, the logistic modelling is insufficient, and survival analysis modelling was therefore chosen for the analysis. To assess the hazard ratio of each type of cancer comparing tattoo exposed to non-exposed, we applied Cox regression with age as timescale and stratification by sex. Tattoo exposure was included as a time-dependent covariate, meaning that the exposure status of an individual was allowed to change over time, i.e. it changed at time of first tattoo. Thus, an individual acquiring their tattoo after being diagnosed with cancer, contributed risk time only as unexposed. Smoking was added as a risk factor to block potential unmeasured confounders such as lifestyle factors associated with smoking. It was defined as a time-dependent covariate, with smoking status changing from non-smoker to smoker at the age at which the individual started smoking. This will be referred to as never smoker vs. ever smoker. We further examined the influence of exposure to certain colors of tattoo ink by comparing the hazard of cancer between individuals with tattoos and those without, while including a term for tattoos without a given color of ink vs. no tattoo as a confounder. Similarly, we assessed a dose-response relationship by splitting tattoo exposure into small and large (large defined as larger than the palm of one’s hand).

We aimed to estimate a measure of association for tattoo exposure and adult cancer occurrence (diagnosed from age 20 years). Therefore, the minimum entry age was set to 20 years. Time of censoring was defined as age on January 1, 2017 (end of follow-up on cancer data). In case of multiple diagnoses of the same type, we only considered time to first diagnose, thus disregarding subsequent diagnoses. Scaled Schoenfeld residuals were applied to assess the proportional hazards assumption. Robust sandwich estimation was applied to account for within-pair dependency for twin pairs. As sensitivity analysis, we carried out the same analyses using a frailty model (using gamma-distributed frailties) to assess the influence of different within-pair dependencies for monozygotic, same-sex dizygotic, and opposite sex dizygotic twin pairs. Also, as sensitivity analysis we applied inverse probability weight (IPW) adjustment for population representativeness of age and sex in the analysis of the twin cohort, as described in [4, 25].

Finally, we did a matched case-cotwin analysis (also known as a discordant twin design analysis) using a stratified Cox model with twin pair-specific baseline hazards. This is a strong design that enables control for unobserved, shared confounding including genetic background and shared environmental factors, such as upbringing [26]. However, as this design is sensitive toward non-shared confounding, it was accompanied by an individual analysis, which is more robust toward non-shared confounding [27].

All analyses were performed using the statistical software *R* version 4.3 [28]. The time-to-event analyses were done using the R packages *survival* [29, 30], *timereg* [31, 32], and *mets* [33, 34].

## Results

### Descriptives

Descriptives of the case-cotwin study are displayed in Table 1a. There were 32 lymphoma cases and 34 cotwin controls (from 32 lymphoma-discordant twin pairs and 18 incomplete twin pairs). The cases included seven individuals that were tattooed before getting diagnosed with cancer, and the median number of years from tattoo to cancer was 8 years (IQR: 4–17 years). Out of the 17 pairs where both twins participated in the survey and provided information on tattoo exposure, fewer than five twin pairs were informative (discordant for both lymphoma outcome and tattoo exposure before time of diagnosis in case twin).

**Table 1a:** Characteristics of the case-cotwin samples of twin pairs with lymphoma, skin, and bladder cancer

		Lymphoma		Skin cancer		Bladder cancer	
		Case	Control	Case	Control	Case	Control
Birth cohort		1960–1986	1960–1993	1960–1991	1960–1990	1960–1979	1960–1984
Median age at follow-up (IQR), years		49 (43–53)	51 (44–55)	49 (41–54)	49 (41–53)	55 (54–56)	52 (48–55)
Individual twins, *n*		32	34	119	104	10	20
Females, *n* (%)		11 (34)	19 (56)	73 (61)	62 (60)	< 5	9 (45)
Tattoos, *n* (%)		10 (31) ^(a)^	11 (32)	30 (25) ^(a)^	25 (24)	< 5	< 5
	Median age at first tattoo (IQR), years	28 (26–36)	22 (18–27)	24 (18–32)	24 (18–37)	-	-
	Tattoos with black ink, *n*	9	10	29	24	< 5	< 5
	Tattoos with red ink, *n*	6	6	11	9	< 5	< 5
	Large tattoos ^(b)^, *n*	7	7	11	6	< 5	< 5
Ever smoker, *n* (%)		13 (41)	13 (38)	54 (45)	37 (36)	5 (50)	9 (45)

^(a)^ Number of these individuals that were tattooed before getting diagnosed with cancer: lymphoma 7 and skin 27

^(b)^ The combined area of the body covered by tattoos was greater than the size of the palm of one’s hand

There were 119 cases among the twin pairs with skin cancer (including 4 concordant twin pairs) and a total of 104 controls (from 67 skin cancer-discordant twin pairs and 44 incomplete twin pairs). The cases included 27 individuals that were tattooed before getting diagnosed with cancer, and the median number of years from tattoo to cancer was 14 years (IQR: 5–20 years). Out of the 71 pairs where both twins participated in the survey, 14 twin pairs were informative. Among these, there were 8 pairs in which the case was exposed while the cotwin was unexposed. Among the twin pairs with cancer of the bladder and urinary tract, there were only 8 cases (all from discordant pairs) and 6 controls. So, there were not enough informative pairs to provide these counts.

Descriptives of the twin cohort study are displayed in Table 1b. Among the 2,367 individual twins, there were 6 cases with lymphoma, 16 with skin cancer (half of them were tattooed with a median number of years from tattoo to diagnosis of 11 years (IQR: 4–20 years)), and 29 with basal cell carcinoma (11 of them were tattooed with a median number of years from tattoo to diagnosis of 14 years (IQR: 7–24 years)). There were 10 individuals among the controls for whom smoking data was missing (< 1%). Out of the 673 pairs where both twins participated in the survey and provided information on tattoo exposure, fewer than five twin pairs were informative for basal cell carcinoma. The number of pairs informative for lymphoma and skin cancer are not included here, as these pairs represent a subset of the case-cotwin samples.

**Table 1b:** Characteristics of the twin cohort split into cases and controls for lymphoma, skin cancer, and basal cell carcinoma

	Lymphoma		Skin cancer		Basal Cell Carcinoma	
	Case	Control	Case	Control	Case	Control
Birth cohort	1961–1985	1960–1996	1960–1991	1960–1996	1960–1980	1960–1996
Median age at follow-up (IQR), years	50 (44–53)	42 (30–50)	50 (46–54)	42 (30–50)	52 (49–55)	41 (29–50)
Individual twins, *n*	6	2,361	16	2,351	29	2,338
Females, *n* (%)	1–5	1,372 (58)	10 (62)	1,367 (58)	19 (66)	1,358 (58)
Tattoo, *n* (%)	< 5	664 (28)	8 (50) ^(a)^	659 (28)	11 (38) ^(a)^	656 (28)
Ever smoker, *n* (%)	< 5	858 (36)	9 (56)	852 (36)	8 (28)	853 (37)

^(a)^ Less than five of these individuals acquired their first tattoo after getting diagnosed with cancer

In addition to Tables 1a and 1b, information on zygosity and median age at smoking initiation is provided in Supplementary Tables 1a and 1b. Furthermore, Supplementary Tables 2a and 2b display the descriptives of all individuals selected for both the case-cotwin studies and the cohort studies, and who were invited to participate in the survey.

The tables include both twins of complete and incomplete twin-pairs.

The individuals presented in Tables 1a and 1b are included in the individual-level analyses while the informative pairs are considered in the matched analyses. We note that only cases tattooed before getting diagnosed with cancer provide risk time as tattoo exposed in these time-to-event analyses. It will not make sense to perform a two-by-two table analysis of counts in Tables 1a and 1b as the follow-up time is not the same among cases and controls.

### Time-to-event analysis

#### The case-cotwin study

The hazard ratio of lymphoma for large tattoo exposure compared to no tattoo was estimated to 2.73 (95% CI: 1.33–5.60) using an individual-level analysis (Table 2). When size was ignored, no evidence of a tattoo effect on hazard of lymphoma could be detected. There was not enough variation to study the effect of tattooing with black ink, and no effect of tattooing with red ink was found. It was not possible to fit a model with smoking as covariate or to fit a matched model for lymphoma.

**Table 2:** Results from individual analysis in case-cotwin study

	Lymphoma		Skin cancer	
	HR (95% CI)	*p*	HR (95% CI)	*p*
Model 1: Tattoo	1.35 (0.68–2.71)	0.39	**1.62** (1.08–2.41)	0.019
Model 2: Tattoo size				
Small	0.63 (0.13–2.94)	0.55	1.37 (0.87–2.16)	0.18
Large	**2.73** (1.33–5.60)	0.006	**2.37** (1.11–5.06)	0.025
Model 3: Red ink				
Yes ^(a)^	1.36 (0.57–3.29)	0.49	1.44 (0.85–2.45)	0.18
No ^(b)^	1.33 (0.36–4.90)	0.67	**1.74** (1.02–2.96)	0.041
Model 4:				
Tattoo	-		**1.68** (1.13–2.51)	0.010
Smoking	-		0.88 (0.62–1.25)	0.47

^(a)^ Red ink in tattoo vs. no tattoo

^(b)^ Tattoo without red ink vs. no tattoo

Hazard ratio of cancer diagnosed from age 20 years among twins born since 1960. Case-cotwin study. Individual-level analysis. Stratification by sex has been applied in all models

The hazard ratio of skin cancer for tattoo exposure was estimated to 1.62 (95% CI: 1.08–2.41) in individual-level analysis (Table 2). When considering size of tattoo, there was evidence of an effect of large tattoos, HR: 2.37 (95% CI: 1.11–5.06). No effect of exposure to red ink in a tattoo could be detected (HR: 1.44 (95% CI: 0.85–2.45)). There was no evidence of a confounding effect of smoking (ever vs. never smoker). The effect of tattoo exposure on hazard of skin cancer in the matched analysis showed HR: 1.33 (95% CI: (0.46–3.84)) and was based on 14 twin pairs discordant for tattoo ink exposure and skin cancer outcome.

It was not possible to estimate the effect of tattooing on hazard of cancer of the bladder and urinary tract.

#### The twin cohort study

The hazard ratio of skin cancer for tattoo exposure was estimated to 3.91 (95% CI: 1.42–10.78) indicating an increased hazard among tattooed individuals (Table 3). Likewise, the hazard ratio of basal cell carcinoma for tattoo exposure was estimated to 2.83 (95% CI: 1.30–6.16). When combining the two cancer types, the hazard ratio was 3.28 (95% CI: 1.76–6.09). There was no evidence of an effect of smoking (ever vs. never smoker) when studying skin cancer outcome. For basal cell carcinoma, the effect of tattooing was estimated to HR = 3.52 (95% CI: 1.63–7.61) when adjusting for smoking. It was not possible to estimate the effects of tattoo exposure on hazard of lymphoma or to do matched case-cotwin analysis.

**Table 3:** Results from individual analysis in twin cohort study

	Skin cancer		Basal cell carcinoma	
	HR (95% CI)	*p*	HR (95% CI)	*p*
Model 1: Tattoo	**3.91** (1.42–10.8)	0.009	**2.83** (1.30–6.16)	0.009
Model 2:				
Tattoo	**3.58** (1.27-10.0)	0.016	**3.52** (1.63–7.61)	0.001
Smoking	1.44 (0.53–3.95)	0.48	0.40 (0.18–0.89)	0.025

Hazard ratio of cancer diagnosed from age 20 years among twins born since 1960. Cohort study. Individual-level analysis. Stratification by sex has been applied in all models

### Sensitivity analysis

Sensitivity analysis indicated no influence of different within-pair dependency for monozygotic, same-sex dizygotic, and opposite sex dizygotic twin pairs. Besides, there were no considerable differences when including inverse probability weight adjustment for population representativeness of age and sex in the twin cohort study. Model assumptions (e.g. proportional hazards) were not found violated in any application.

## Discussion

In the case-control study, individual-level analysis resulted in a hazard of skin cancer that was 1.62 times higher among tattooed than non-tattooed participants (95% CI: 1.08–2.41). The twin-matched analysis of 14 twin pairs discordant for tattoo ink exposure and skin cancer shows HR = 1.33 (95% CI: 0.46–3.84). For both skin cancer and lymphoma, increased hazards were found for tattoos larger than the palm of a hand: HR = 2.37 (95% CI: 1.11–5.06) and HR = 2.73 (95% CI: 1.33–5.60), respectively. In the cohort study design, individual-level analysis resulted in increased hazards among tattooed for skin cancer, HR = 3.91 (95% CI: 1.42–10.8), and basal cell carcinoma, HR = 2.83 (95% CI: 1.30–6.16).

Larger tattoos may show a stronger effect either due to higher dose of exposure or longer time of exposure (from tattoos acquired over time). Likewise, absence of red ink shows an effect; however, some ambiguity arises due to color effects, as many colors are typically present at the same time. We are cautious about interpretation and recommend more detailed studies of size and color.

The case-control sample of twin pairs with cancer of the bladder and urinary tract expectedly did not contain enough cases to study the effect of tattoo ink exposure. It is possibly too early to study this association since bladder cancer mainly occurs at high ages, that is, among individuals from generations where tattoo prevalence is currently low [4].

Our study was initiated based on the suspicion that ink deposits will interact with surrounding tissue causing increased cell proliferation and thereby increase cancer risk. We term this *the ink deposit conjecture*. The mechanism involves an immunologic response and is recognized, for instance, in breast implant-associated anaplastic large cell lymphoma (BIA-ALCL) – a rare type of T-cell lymphoma [35]. We stress that this pathway does not necessarily involve specific ink agents; however, if carcinogenic compounds are present, the pathway is expectedly different but still leads to increased cancer risk. Consequently, the preventive effects of the recent European restrictions [36] intended to limit exposure to a long list of known or suspected carcinogenic compounds may be lower than first anticipated. Our findings are consistent with the conjecture and with reported findings, such as squamous cell carcinoma, benign tumors, lymphoid conditions, and rare cases of malignant neoplasms occurring within the area of a tattoo [37–42].

Twins per se are highly representative of the general population. There is solid evidence that being a twin does not influence the risk of cancer. The incidence of cancer among twins has been demonstrated to mirror that of the general population [43, 44].

In the Nordic countries, individuals born more recently do not have markedly higher age-specific incidence rates of non-Hodgkin lymphoma and non-melanoma skin cancer, thus not supporting a major role of tattoos for cancer incidence in the population [23, 24]. It may be that the proportion of cases accounted for by the increase in incidence for tattoos is too small to be detected in the overall variation of cancer incidence. For skin cancer, it may be balanced out by e.g. increasing use of methods and behaviors to reduce sun exposure.

As of June 2024, to the best of our knowledge, there are only three publications in the field. The first is a study from 2020 about cosmetic tattooing and early onset basal cell carcinoma in New Hampshire [21]. The study sample was based on a matched case-control design, but only exposed individuals (156 tattooed cases and 213 tattooed controls) were included in the analysis. They compared odds of being tattooed within the “anatomical region” of the basal cell carcinoma (as opposed to being tattooed at a different site) to odds of being tattooed within randomly assigned “reference sites” and found an odds ratio of 1.8 (95% CI: 1.0-3.2) hinting toward an association. The second study is a Canadian study from 2020 [19] that considered two population-based case-control studies holding 1,518 participants (737 cases) from a study of non-Hodgkin lymphoma and 742 (373 cases) from a study of multiple myeloma. Using logistic regression modelling, they found no association with tattoo exposure. The third study, a Swedish population-based case-control study of lymphoma from 2024 [20], included 1,398 cases and 4,193 controls identified through incidence density sampling. The main result of an increased risk of lymphoma among tattooed was borderline significant. Through conditional and unconditional logistic regression (i.e. matched and unmatched analysis) using basic and extended confounder adjustment, they estimated four incidence rate ratios (IRR) of lymphoma of which three were statistically significant, thus providing evidence of increased risk among tattooed individuals. For the matched analysis, they found IRRs of 1.21 (95% CI: 0.99–1.48) and 1.24 (95% CI: 1.02–1.50) for models with extended and basic confounder adjustment, respectively. A strength of the study is that it obtained very similar IRRs in both unadjusted and adjusted as well as matched and individual-level analyses. The authors discussed estimates relating to dose-response relationships and influence of exposure time; however, the results presented provided no evidence to support this discussion.

The strength of applying two designs of studies (individual and matched) with complementary advantages allowing for extended confounder control is also explored in the present study. The twin sample provides a highly valid and representative control group, e.g. in terms of age, sex, upbringing, and genetic similarity. Additional merits of our study are: (i) The application of time-to-event analysis using age as time scale to precisely specify risk time according to age of being tattooed or non-tattooed at time of diagnosis, thus avoiding immortal time bias and bias from incomplete follow-up. In general, considering a time-varying covariate as constant typically leads to underestimation of exposure effect. (ii) Using inverse probability weights for age and sex representativeness for better confounder adjustment (compared to standard covariate adjustment).

Our sample consists of cancer survivors participating in the survey. Those dying from e.g. severe cancer will not be represented. This group consists of less than 10% of the eligible twins who also included emigrated individuals and those waivered contact by researchers. We suspect limited survivorship bias.

There are several different types of tattoos. The intended focus of this study was on “classic” decorative tattoos. However, since the survey did not specify type of tattoo to the participants, individuals with permanent make-up (PMU) and medical tattoos may also have responded as being tattooed. In hindsight, there should have been a question distinguishing these types of tattoos as they generally differ in both size and type of ink used.

When assessing the influence of smoking, the time-varying covariate was defined only from age when the participants started smoking and assumed one never stopped smoking. Here only crude smoking information was included, and we did not include e.g. number of pack years or, in a matched analysis, by considering the total number of years as a smoker until time of diagnose in the case twin.

The lack of information on sun exposure in the present study is a limitation. While there may be a confounding effect, the direction is ambiguous. It is hypothesized that tattooed individuals are more prone to sun-exposing behavior while showing off their tattoos. On the other hand, they may also be less prone to expose their tattoos to the sun to avoid photodecomposition. A recent study suggests that tattooed (non-European) individuals are more exposed to the sun, but also display more sun-protection habits [45].

Association between tattoo ink exposure and skin cancer outcome may be the result of later detection due to abnormalities being concealed by the ink. In other words, tattoo ink may not cause cancer but ‘merely’ lead to later detection and thus potentially be associated with more severe stages of skin cancer. This is something we plan to delve deeper into in the future in studies of pathogenesis of certain subtypes of skin cancer.

Another limitation of the study is the exclusion of basal cell carcinoma in the case-cotwin study. The finding of an association between tattoo ink exposure and basal cell carcinoma from the cohort study would have been strengthened if similar results were obtained in the case-cotwin study. This was not possible, however, since basal cell carcinoma was not among the selected cancer types for which twins were invited to participate in the survey.

Besides, the present study was restricted to individuals born since 1960 to avoid bias from birth cohort differences. However, the Danish Twin Tattoo Cohort holds information dating back to the time of the opening of the Danish Cancer Register in 1943. Thus, it would be possible to extend the follow-up period, but alternative means of analysis would be required. It is difficult to model association between tattoo exposure and cancer incidence over such a long period because (i) the popularity of tattooing has increased markedly in recent decades [4] and (ii) cancer diagnostic procedures have improved markedly over the years. Additionally, the incidence of both tattooing and cancer varies with age. That is, two timescales are in play: individual age and calendar time. Through Poisson modelling it is possible to estimate a hazard ratio of cancer by tattoo exposure while incorporating both timescales. Also, to enable matched case-cotwin modelling, (expectedly the strongest possible design for studying exposure-outcome association), larger cohorts are needed. There is a potential to expand the Danish Twin Tattoo Cohort to include the other Nordic countries through their respective twin registers.

Additionally, having a tattoo, especially among adolescents, has been suggested as an indicator of risky lifestyle highly associated with e.g. smoking [4] and alcohol consumption [46] – both risk factors of certain cancer types. Hence, evidence of an association between tattoo ink exposure and occurrence of cancer may be confounded by other health-related lifestyle factors. We intend to exploit the remainder of the information gathered in the survey in the future – both regarding lifestyle factors, but also the tattoo details, e.g. is it possible to point at one specific color of tattoo ink? Additionally, there is potential for updated follow-up and extension of the project in the future. For instance, through linkage with national disease registers, one could study the association between tattoo ink exposure and incidence of other diseases of the immune system.

Besides, there are other concerns related to long-term safety of tattooing that remain to be studied. With the increasing popularity of getting tattooed, it is necessary to question the safety of laser tattoo removal where pigments are broken into smaller fragments that leave the site of the tattoo. The question is: Where do the pigment fragments end up? Decreasing particle size often allows for greater migration potential [11]. This is also an issue in relation to decomposition of ink particles induced by sun radiation [15]. Besides, with ink particles travelling through the blood, could tattooing during pregnancy or the following period of breast feeding be injurious to the health of the offspring? Furthermore, on the more speculative side, we suggest research into ink compositions that can be dissolved or investigations into medical treatments that can remove some of the persistent chemicals from the body, especially the lymphatic system.

A public health policy to prevent tattoo ink-induced carcinogenesis would be to be cautious until further knowledge is obtained. As demonstrated in a recent study of the Danish Twin Tattoo Cohort, the decision of becoming tattooed is strongly driven by environmental influences, while genes have very limited influence [4]. Consequently, public health interventions such as informative campaigns would expectedly be among the most effective preventive measures against cancers caused by tattooing.

In conclusion, our study points toward increased hazard of lymphoma and skin cancers among tattooed individuals, demonstrated through two designs: a twin cohort and a case-cotwin study. We are concerned that tattoo ink has severe public health consequences since tattooing is abundant among the younger generation. Studies that pinpoint the etiological pathway of tattoo ink-induced carcinogenesis are recommended.

## Electronic supplementary material

Below is the link to the electronic supplementary material.

Supplementary Material 1

## Data Availability

Restrictions apply to the availability of these data. Requests to access data need to be made through the Danish Twin Registry.

## Abbreviations

DTR: Danish Twin Registry
DTTC: Danish Twin Tattoo Cohort
HR: Hazard ratio
IARC: International Agency for Research on Cancer
IPW: Inverse probability weights
IQR: Interquartile range
