# Supplementary material for: Tattoo ink exposure is associated with lymphoma and skin cancers – a Danish study of twins
Source: BMC Public Health. 2025 Jan 15;25:170. doi: 10.1186/s12889-025-21413-3 (PMC11736920; doi:10.1186/s12889-025-21413-3)
Supplement: Supplementary file 1 — Supplementary Material 1 [file 12889_2025_21413_MOESM1_ESM.docx]

# Supplementary materials

**Title**: Tattoo ink exposure is associated with lymphoma and skin cancers – a Danish study of twins

**Journal**: BMC Public Health

**Authors**:

Signe Bedsted Clemmensen^1,2,^* (<https://orcid.org/0000-0001-9871-149X>), Jonas Mengel-From^1,2,3^ (<https://orcid.org/0000-0003-1573-8908>), Jaakko Kaprio^4^ (<https://orcid.org/0000-0002-3716-2455>), Henrik Frederiksen^5,6^ (<https://orcid.org/0000-0001-8905-0220>), Jacob von Bornemann Hjelmborg^1,2^ (<https://orcid.org/0000-0001-9630-9149>).

^1^ Department of Epidemiology, Biostatistics, and Biodemography, Institute of Public Health, University of Southern Denmark, Odense, Denmark.

^2^ Danish Twin Registry, Institute of Public Health, University of Southern Denmark, Odense, Denmark.

^3^ Department of Clinical Genetics, Odense University Hospital, Odense, Denmark.

^4^ Institute for Molecular Medicine Finland FIMM, HiLIFE, University of Helsinki, Helsinki, Finland.

^5^ Department of Haematology, Odense University Hospital, Odense, Denmark.

^6^ Department of Clinical Research, University of Southern Denmark, Odense, Denmark.

* Corresponding author: [sbclemmensen@health.sdu.dk](mailto:sbclemmensen@health.sdu.dk)

**Supplementary Figure 1.** Overview of the questionnaire used in the Danish Twin Tattoo Cohort.


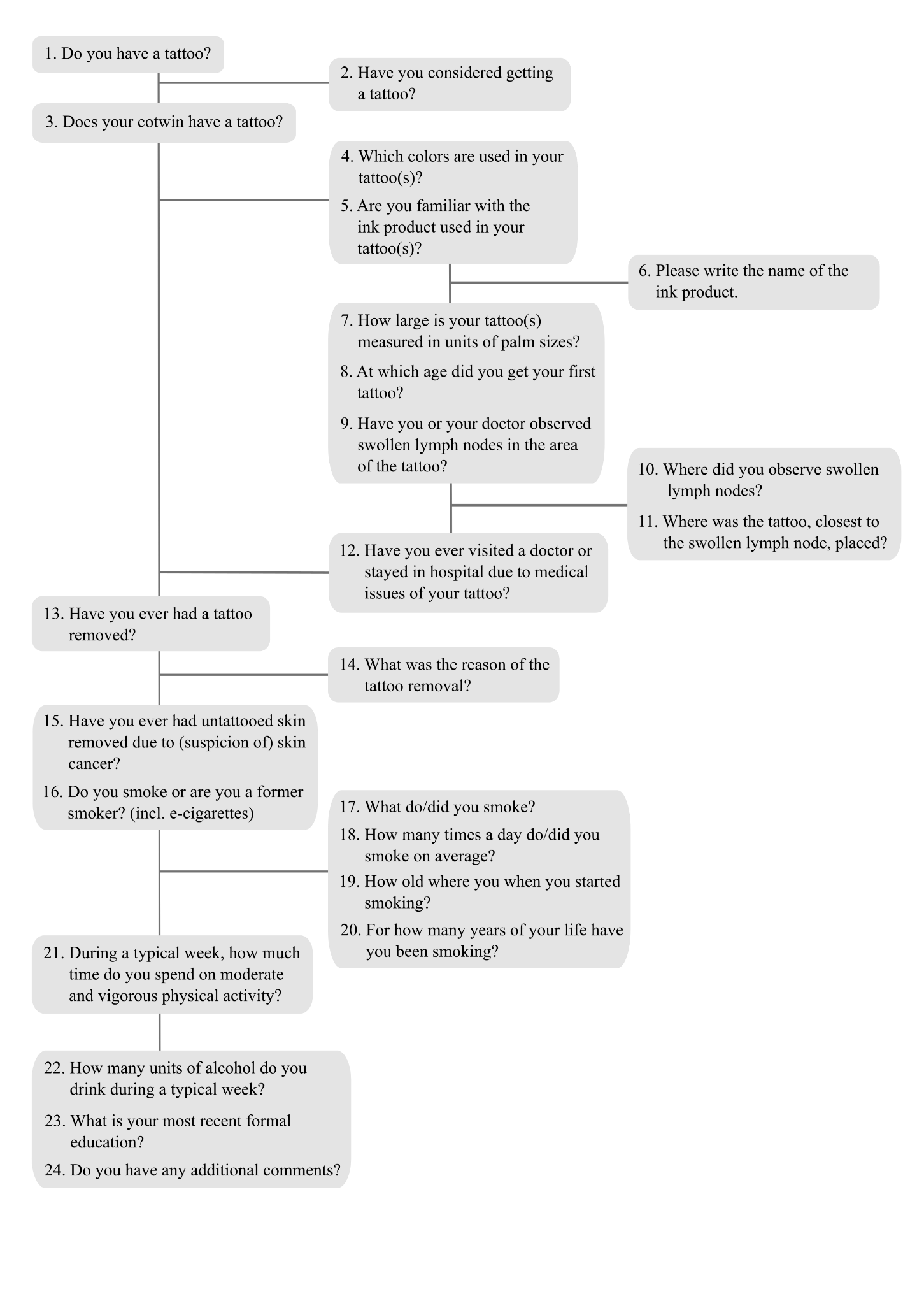


**Supplementary Table 1a.** Characteristics of the case-cotwin samples of twin pairs with lymphoma, skin, and bladder cancer.

|  | Lymphoma | | Skin cancer | | Bladder cancer | |
| --- | --- | --- | --- | --- | --- | --- |
|  | Case | Control | Case | Control | Case | Control |
| Zygosity, n (%) |  |  |  |  |  |  |
| MZ | 5 (16) | 8 (24) | 36 (30) | 34 (33) | <5 | 7 (35) |
| DZ | 12 (38) | 14 (41) | 45 (38) | 41 (39) | 5 (50) | 5 (25) |
| OS | 15 (47) | 12 (35) | 38 (32) | 29 (28) | <5 | 8 (40) |
| Median age at smoking initiation (IQR), years | 17 (15-19) | 16 (14-18) | 17 (15-18) | 16 (15-17) | 15 (14-15) | 17 (16-17) |

Additional to Table 1a.

**Supplementary Table 1b**. Characteristics of the twin cohort split into cases and controls for lymphoma, skin cancer, and basal cell carcinoma.

|  | Lymphoma | | Skin cancer | | Basal Cell Carcinoma | |
| --- | --- | --- | --- | --- | --- | --- |
|  | Case | Control | Case | Control | Case | Control |
| Zygosity, n (%) |  |  |  |  |  |  |
| MZ | <5 | 636 (27) | <5 | 632 (27) | 11 (38) | 626 (27) |
| DZ | <5 | 866 (37) | 9 (56) | 861 (37) | 11 (38) | 859 (37) |
| OS | <5 | 859 (36) | <5 | 858 (36) | 7 (24) | 853 (36) |
| Median age at smoking initiation (IQR), years | - | 16 (14-18) | 17 (17-25) | 16 (14-18) | 16 (14-17) | 16 (14-18) |

Additional to Table 1b.

**Supplementary Table 2a**. Characteristics of invited twin pairs in case-cotwin study.

|  | Lymphoma | | Skin cancer | | Bladder cancer | |
| --- | --- | --- | --- | --- | --- | --- |
|  | Case | Control | Case | Control | Case | Control |
| Birth cohort | 1960-1996 | 1960-1996 | 1960-1994 | 1960-1994 | 1960-1984 | 1960-1984 |
| Median age at follow-up (IQR), years | 49 (43-53) | 49 (43-54) | 48 (41-53) | 48 (40-53) | 53 (49-55) | 53 (48-55) |
| Individual twins, n | 48 | 57 | 182 | 175 | 24 | 24 |
| Females, n (%) | 17 (35) | 31 (54) | 109 (60) | 100 (57) | <5 | 10 (42) |
| Zygosity, n (%) |  |  |  |  |  |  |
| MZ | 8 (17) | 12 (21) | 51 (28) | 53 (30) | 7 (29) | 9 (38) |
| DZ | 19 (40) | 21 (37) | 73 (40) | 66 (38) | 11 (46) | 7 (29) |
| OS | 21 (44) | 24 (42) | 58 (32) | 56 (32) | 6 (25) | 8 (35) |
| Survey participation, n (%) | 32 (67) | 34 (60) | 119 (65) | 104 (59) | 10 (42) | 20 (83) |

Characteristics of the case-cotwin samples of twin pairs invited to participate in the survey where at least one twin in a pair was diagnosed with lymphoma, skin, or bladder cancer.

**Supplementary Table 2b**. Characteristics of invited twin pairs in cohort study.

|  | Lymphoma | | Skin cancer | | Basal cell carcinoma | |
| --- | --- | --- | --- | --- | --- | --- |
|  | Case | Control | Case | Control | Case | Control |
| Birth cohort | 1961-1985 | 1960-1996 | 1960-1991 | 1960-1996 | 1960-1993 | 1960-1996 |
| Median age at follow-up (IQR), years | 51 (44-52) | 40 (28-49) | 49 (46-52) | 40 (28-49) | 50 (46-53) | 40 (27-49) |
| Individual twins, n | 10 | 4,522 | 27 | 4,505 | 45 | 4,487 |
| Females, n (%) | 6 (60) | 2,299 (51) | 15 (56) | 2,290 (51) | 26 (58) | 2,279 (51) |
| Zygosity, n (%) |  |  |  |  |  |  |
| MZ | 1-5 | 1,156 (26) | 9 (33) | 1,150 (26) | 17 (38) | 1,142 (25) |
| DZ | 5 (50) | 1,666 (37) | 13 (48) | 1,658 (37) | 17 (38) | 1,654 (37) |
| OS | 1-5 | 1,700 (38) | 5 (19) | 1,697 (38) | 11 (24) | 1,691 (38) |
| Survey participation, n (%) | 6 (60) | 2,362 (52) | 16 (59) | 2,352 (52) | 29 (64) | 2,339 (52) |

Characteristics of the cohort for all twins invited to participate in the survey split into twins with and without lymphoma, skin cancer or basal cell carcinoma.
